# The Impact of Extraversion and Introversion on Millennials Propensity to Recommend Their Preferred Fitness Center

**DOI:** 10.3390/bs14010022

**Published:** 2023-12-27

**Authors:** Dan-Cristian Dabija, Luiela Magdalena Csorba, Nicolae Horațiu Pop, Daniel-Rareș Obadă

**Affiliations:** 1 Department of Marketing, Faculty of Economics and Business Administration, Babeș-Bolyai University Cluj-Napoca, 400591 Cluj-Napoca, Romania; 2 Department of Economics, Faculty of Economic Sciences, Aurel Vlaicu University Arad, 310130 Arad, Romania; luiela.csorba@uav.ro; 3 Department of Individual Sports, Faculty of Physical Education and Sport, Babeș-Bolyai University Cluj-Napoca, 400376 Cluj-Napoca, Romania; nicolae.pop@ubbcluj.ro; 4 Department of Communication Sciences and Public Relations, Faculty of Philosophy and Socio-Political Sciences, Alexandru Ioan Cuza University Iași, 700506 Iași, Romania; daniel.obada@uaic.ro

**Keywords:** Millennials, extraversion, introversion, fitness center, satisfaction, propensity, recommendation of the fitness center, image of the fitness center, generational theory, SmartPLS

## Abstract

Millennials of the 21st century tend to have an active daily life and practice more sports, spending more on health and fitness. Therefore, the present paper seeks to investigate the propensity of Millennials to recommend their preferred fitness center, depending on their psychological condition (i.e., introversion versus extraversion), which may lead to their tendency to practice and to their involvement and satisfaction with the fitness center. The authors deduce a conceptual model from the literature, which is further analyzed with data collected through an empirical investigation based on a face-to-face administered questionnaire. Data gathered from 2138 Millennials are investigated with the help of structural equation modeling using SmartPLS. The results show that a positive image of a fitness center stimulates the satisfaction of extravert and introvert consumers. Millennials have different personality types, such as extraversion and introversion, that influence their propensity to practice fitness and to recommend their favorite fitness center. It can be concluded that not only extraversion but also introversion influence consumers’ satisfaction, positively impacting the consumer’s intention to recommend their favorite fitness center to peers.

## 1. Introduction

As individuals in contemporary society can enjoy lots of interesting activities without even leaving their homes, they also tend to be more sedentary and disregard, or even avoid, sporting activities. Modern life involves stress, a sedentary lifestyle, and a lot of physical fatigue [1]. People’s free time and time for socializing are increasingly reduced. Despite this, there is a growing emphasis on health promotion and disease prevention, particularly for older adults, as a means of enabling them to live longer and healthier lives. The promotion of healthy lifestyles in modern society is a multifaceted issue with medical, social, and spiritual implications [2]. Extensive scientific and medical evidence underscores physical inactivity as a major public health issue, contributing to over 50% of health problems. Exercise, recognized for treating and preventing chronic conditions, faces a compliance gap despite physicians advice to patients [3]. However, there is a gap between the global value of health and its practical implementation, with the concept of a healthy lifestyle often being idealized and not fully realized in daily practices [4]. A range of healthy lifestyle products is also available, including a huge assortment of dietary and nutritional supplements [1,5]. Physical culture, sports, and healthy lifestyle interconnection are also emphasized. For a healthy lifestyle, the need for physical activity, either indoors or out in nature, has increased in importance. Practicing fitness has become increasingly important for different generations, especially young individuals who dedicate themselves to sports [6]. Fitness centers are popular among the younger generation due to their focus on customer satisfaction and loyalty [7]. For example, in Romania, an emerging market, the number of fitness centers increased from 277 in 2018 to 1699 in 2020 [8]. These centers provide a controlled environment for physical activity, which can improve both physical and mental well-being by offering a space for social interaction [9]. Fitness centers not only foster social connections and offer diverse activities but also provide professional guidance, cutting-edge equipment, time-efficient options, motivational atmospheres, comprehensive health programs, and technological integration, creating an engaging and holistic exercise experience.

In a digital society, the young generation needs more socialization and the feeling of belonging to a social group, which is why fitness centers play a special role in their lives [10]. For example, Millennials are a group of consumers who care about their health, personal image, and personal beauty and who wish to build their self-esteem, especially through their public appearance [5]. Moreover, care for health and body aesthetics leads them to purchase not only the services offered by the fitness center but also various natural products (vitamins, energy drinks, and other dietary supplements) [1,5]. The literature also highlights the impact of social media on Millennials’ fitness behavior [11], as well as their concern to adopt new technologies while practicing fitness exercises [12]. A literature analyzing Millennials’ involvement in fitness exercises hardly exists; research is scarce regarding Millennials’ propensity towards practicing fitness depending on their psychological typology (introverts versus extraverts) as well as their involvement with fitness centers.

For fitness center providers, it is essential to know whether the clients are satisfied with the quality of the services offered and if they are disposed toward further recommending the fitness center to peers [5]. Therefore, to investigate how Millennials’ psychological type (introversion versus extraversion) affects their willingness to recommend their favorite fitness center, which, in turn, affects their level of involvement and satisfaction with fitness centers, an empirical study was conducted in a new market where fitness is popular among young people.

The paper extends previous knowledge based on generational theory [13,14], which suggests that, depending on their birth year, individuals can be categorized into different generations. While older customers belong either to “Baby Boomers” or “Generation X”, younger fitness enthusiasts are considered “Millennials”/“Generation Y” or “Generation Z” [15]. This investigation only focuses on Millennials, individuals born between 1981 and 1996 [15]. Their behavior towards fitness centers depending on their psychological condition, extraversion versus introversion, is investigated [16]. Extravert and introvert are two personality types further analyzed in Myers–Briggs psychological theory [17], an adaptation of Jung’s theory [16]. While extraverts are sociable, friendly, excitable, and enjoy being the center of attention, introverts are passive, reserved, shy, and more likely to spend time alone [18]. Extraversion has been correlated with fitness practice [19], but, to the best of our knowledge, there are no previous studies linking introversion and extraversion to Millennials’ propensity towards practicing fitness or recommending their preferred fitness center to their peers. Because the two sides of the human personality are determining factors in Millennials’ behavioral intentions [20,21], especially in terms of their intention to recommend their favorite fitness center to peers, we consider that this issue is worth investigating.

The novelty of this research lies in consideration of the effect of the two psychological conditions (introversion versus extraversion) for Millennials in an emerging market and in highlighting several related aspects of individuals’ fitness center involvement and satisfaction with the fitness center, as well as the perceived image of their preferred fitness center and their propensity towards recommending it to peers. It is interesting to examine if the personality and consumer characteristics of the young generation/Millennials and their need to practice fitness would influence their behavior towards recommending their favorite fitness center to peers, to improve the image of the company, and implicitly to attract new customers, thus supporting the efforts of the fitness services provider.

The paper is structured as follows: The introductory section is followed by a review of the literature and the development of the hypotheses. The third section consists of the research methodology, leading to the findings and discussions.

## 2. Literature Review

### 2.1. Generational Theory

Generational theory is based on the generational cohorts concept proposed in 1952 [22] and further developed in 2013 [23]. A generational cohort refers to several individuals who were born, grew up, and were influenced by the same economic, social, political, cultural, and situational context, including exposure to all kinds of crises, together creating the basis for their grouping in collectives that share many common features [24]. The members of a generational cohort share common values, react to the same stimuli, feel the same emotions, and have similar experiences [25].

Generational cohort theory was subsequently developed by Inglehart [26], who divided the world population into generational cohorts, grouped according to their birth year. One cohort extends across a period of approximately 15 years, with individuals born during that period sharing similar ideas, attitudes, beliefs, values, moral codes, and experiences [27]. These lead to the development of generational identity, meaning that individuals demonstrate specific purchasing behaviors and/or lifestyles [28,29,30]. Even if the ideology of a cohort exists globally, it is interpreted according to each national context, creating cross-cultural similarities and discrepancies when producing a consumer’s profile [31]. Marketing seeks to understand the motivation of each generational cohort concerning the way they interact with and prefer particular companies, brands, and/or different products and services [32,33]. The behavior and psychology of each generational cohort generate lifelong effects [34]. Managers need to develop appropriate marketing strategies depending on the desires, expectations, and needs of each generation, meaning that they must better understand the psychological profile of each consumer typology.

Consumer generations are divided according to their birth year into “Baby Boomers” (born between 1945 and 1964), “Generation X” (born between 1965 and 1979/1980), “Millennials” or “Generation Y” (born between 1980/1981 and 1994/1995), “Generation Z”/“iGeneration” (born between 1995/1996 and 2010), and “Generation Alpha” (born in 2010) [23,27]. Millennials are considered a postmodern generation, known as “heroes” because they were born after an awakening period with weak social institutions, are more protective and optimistic compared to “Xers”, and also tend to be overprotected by their parents [35]. But regardless of which generation a consumer belongs to [36], consumers are differentiated based on their psychological profiles [37], as well as the social and cultural context and situations they are exposed to during their lifetime. The psychological condition of the consumer plays an important role in deciding whether to purchase a product or service. In choosing to attend a fitness center, Millennials’ choices may be influenced by their personality type (extraversion or introversion) [16], but also by key factors, such as cost, service quality improvement, waiting time, noise level, and service responsiveness [38], as well as the perceived quality and subjective norms of the offered services [39].

The first pair of traits, extraversion and introversion, part of the interpersonal dimension, significantly impact an individual’s social interactions [40]. These traits, extensively studied since 1930 [41,42], are multifaceted, adapting to different situations and behaviors based on context. The interpretation of these traits varies with different taxonomies [42]. According to Jung [16], high extraversion directs energy outward, while high introversion directs it inward. Individuals exist on a continuum between the poles of extraversion and introversion, with characteristics varying along this line. Highly extraverted individuals are socially comfortable, open to new experiences, and interested in the external world, while highly introverted individuals prefer introspection and solitude [42].

Norman’s five factor model [43] describes extraverted individuals as talkative, open, adventurous, and sociable, while introverts are silent, secretive, cautious, and reclusive. Extraversion includes traits like sociability, positive emotions, and personality characteristics such as being affectionate and loving, conversational, fun-loving, and passionate [44,45]. In essence, liking to socialize exemplifies the extraversion trait [46]. Conversely, introversion or social isolation reflects a greater inclination towards the internal world. Individuals with low extraversion levels tend to be shy, reserved, and less adept at socializing across various contexts, often being loners and quieter [44,46]. Fitness centers, as well as wellness relaxation, are daily activities for a healthier lifestyle in the opinion of Millennials, who try to keep in balance a work–life equilibrium [15,47]. That is why they are also open to the use of apps and technology for staying healthy and spending more time and money on health and fitness compared with other generational cohorts.

### 2.2. The Millennials’ Behavioral Intention to Practice Fitness

#### 2.2.1. Extraverted Millennials’ Behavioral Intention

Sports and physical activities have a major role in positively influencing human health and emotional well-being, encouraging mankind to have a more active lifestyle [48]. While some physical activities can be practiced individually, such as going to a fitness center or swimming, others are collective, requiring a counterpart (like tennis) or even a small or large team (football, basketball, rugby, etc.) [49]. Apart from playing in a team, socialization is also relevant in building team spirit. Although socialization is not synonymous with extraversion, extraverted individuals tend to be more sociable. When analyzing human personality facets and individual behavior, feelings, thinking, and acting across different periods or under different circumstances, the big five model can be used as a theoretical framework [21]. This model comprises the five dimensions of human personality: extraversion (being a sum of sociability, enthusiasm, and assertiveness), agreeableness (meaning compassion, forgiveness, and being polite), conscientiousness (being organized, self-disciplined, and industrious), neuroticism (meaning irritability, moodiness, and vulnerability), and finally openness to new experiences (a sum of curiosity, creativity, and artistic behavior) [21]. The first dimension, extraversion, positively influences the exercise level of participants in sports, being the strongest personality trait [50], because extraverts are polite, assertive, forgiving, and more [16,19]. Extraversion also has a favorable impact on individual optimism [51] and on habitual Facebook usage [52]. People who behave in an extraverted manner are more capable of increasing their well-being and happiness [53]. Team sports champions’ extraversion and openness are higher than those in individual sports [54]. Because extraverts and Millennials are more concerned about their health, image, body aesthetics, personal beauty, self-esteem, and public appearance compared with other human personalities [5], they may have stronger positive behavioral intentions, such as, e.g., a higher interest in practicing fitness and in being a member of a fitness club, a higher propensity to recommend the fitness center to peers and to attract new members, etc. Positive behavioral intention can be considered as an attitude that reflects the growing interest of Millennials both in the continuous practice of this sport and in recommending it to peers to support the efforts of the company’s management in attracting new clients. Therefore, the following hypothesis is proposed:

**Hypothesis 1** **(H_1_).** *Extraversion exerts a positive influence on Millennials’ propensity to practice fitness.*

The role of fitness in society is increasingly important, both for humans and for public health [49]. Extraversion is strongly correlated with fitness practice [19]. There is a strong positive relationship between extraversion and leisure motivation, with extravert Millennials being highly motivated to attend fitness centers [55]. The construct of active fitness center involvement can be regarded as the desire of Millennials to be regularly informed about any details relating to their favorite sport, in this case, fitness. This involves gathering information about the sport and about the services offered in a fitness center, from the Internet and from newspapers, magazines, or TV. Furthermore, extraversion is favorably related to the customer–employee relationship [20]. Assuming that extraverts are more interested in being actively involved in gathering new information regarding their favorite sport, the following hypothesis is proposed:

**Hypothesis 2** **(H_2_).** *Extraversion has a positive influence on active fitness center involvement.*

The importance of extraversion in ensuring the well-being of a customer has also been analyzed [56]. There is a positive relationship between extraversion and life satisfaction [57,58]. Extraversion positively influences life quality, generating satisfaction [59]. Extraversion is linked to individual well-being and life satisfaction [60]. Extravert individuals enjoy more interpersonal satisfaction than introvert individuals, with extraversion having a positive impact on customer satisfaction [61]. Extraversion also has an impact on the purchasing intention of a Millennial [62], associated with a high level of satisfaction. Therefore, the next hypothesis is proposed as follows:

**Hypothesis 3** **(H_3_).** *Extraversion generates Millennial’s satisfaction towards the fitness center.*

#### 2.2.2. Introverted Millennials’ Behavioral Intention

Individuals have different personalities, some of them falling into the extravert category and others into the introvert category. Based on international statistical data, introverts represent between 33% and 50%, although there is limited information regarding introversion compared with extraversion in the relevant literature [63]. Sport, here, the use of the services offered by a fitness center, has positive effects on the customer’s health [64], more precisely on the body, mind, and emotional equilibrium [65]. Introverted people are reserved individuals preferring a quiet environment, being anxious and inhibited [63]. Because of their personality characteristics, individuals who behave in an introverted manner are more likely to decrease their well-being [53]. That is why, for individuals who prefer to spend time alone, working out at a fitness center makes them feel good and stronger. Indeed, introversion has a positive impact on Millennials’ purchase intentions [62]. In consequence, the next hypothesis can be proposed:

**Hypothesis 4** **(H_4_).** *Introversion favorably influences Millennials’ propensity to practice fitness.*

The degree of satisfaction experienced by a consumer as a result of the consumption of a good or service influences their behavioral intention [66]. That is why customer satisfaction is a predictor of purchase intention [67]. Extraverted individuals feel higher satisfaction with their social relationships compared to introverts [68,69], which has a huge impact on customer loyalty [70]. As we intend to determine whether introversion affects Millennials’ satisfaction with the image and services offered by a fitness center, the following hypothesis is proposed:

**Hypothesis 5** **(H_5_).** *Introversion has a positive influence on Millennial’s satisfaction with the fitness center.*

The attributes of a brand have an impact on the intention of individuals to consume sports services [71]. The image of a fitness center is similar to the brand or product image. If a brand has the power to influence the consumer’s propensity to buy it [72], it can be argued that potentially the image of a fitness center can determine whether young people (e.g., Millennials) will become members. While extraverts are more open to change than introverts (as suggested by the Myers–Briggs psychological theory [17] and Jung’s theory) [16], we are interested in investigating introverts’ position regarding this aspect. The following hypothesis is proposed:

**Hypothesis 6** **(H_6_).** *Introversion has a positive influence on the fitness center’s image.*

#### 2.2.3. The Influence of Extraverted and Introverted Millennials on Strengthening the Fitness Center Image and Active Fitness Center Involvement

Physical activity positively influences consumer health, having a positive effect on the body and the inner equilibrium of human beings [72]. Millennials, regardless of whether we refer to them as extraverts or introverts, are always attracted to offers, especially from companies that offer discounts or incentives [5]. If fitness clubs offer incentives, Millennials are encouraged to increase the time spent in the centers and to do more exercises [73], which means a higher propensity to practice fitness. Furthermore, incentives stimulate the Millennials’ propensity to recommend the fitness center to peers [5]. Both of the aforementioned propensities reflect Millennials’ active fitness center participation. Furthermore, there is a positive relationship between the individual’s need for uniqueness in sport and consumption choice [74], as well as between the consumer’s intention (behavior) to use a fitness center app and the overall satisfaction of the fitness club members [75]. Therefore, it can be postulated that:

**Hypothesis 7** **(H_7_).** *The propensity to practice fitness has a positive influence on active fitness center involvement.*

Fitness exercises play a key role in providing high life quality and mental health [76]. Relationships between the extraverted or introverted Millennials’ propensity to practice fitness, active fitness center involvement, and fitness center image have not been investigated in the literature. The literature suggests only that there is a relationship between the tendency to exercise in a fitness environment and a negative body image, especially in women [77]. As a result, the following hypotheses are proposed:

**Hypothesis 8** **(H_8_).** *The propensity to practice fitness positively influences the fitness center’s image.*

**Hypothesis 9** **(H_9_).** *Active fitness center involvement generates the fitness center image.*

Corporate image influences consumer trust [78] and consumers’ loyalty indirectly, but significantly [79]. The image of a fitness center has a positive influence on customers’ trust [80]. The link between corporate image and consumer loyalty, with consumer satisfaction as a mediator, was previously validated [81,82]. Therefore, the next hypothesis proposed is that:

**Hypothesis 10** **(H_10_).** *The fitness center image positively influences Millennials’ satisfaction with the fitness center.*

A high level of credibility in the services offered by a fitness center positively influences Millennials’ attitudes and future behavior, while the fitness center’s image influences individual recognition, which impacts the customer’s attitude and future intentions [80]. Satisfaction and consumers’ perceived value influence the customer’s future behavioral intentions [83]. Trust in a brand and commitment to the brand in sports events favorably influence the recommendation of the event brand to other participants [84]. While customer trust and satisfaction play the role of mediating variables, corporate image influences the Millennials’ recommendation (word of mouth) of a fitness center [85]. Hence, the following hypotheses are proposed:

**Hypothesis 11** **(H_11_).** *The center’s image has a positive influence on Millennials’ propensity towards recommending the fitness center.*

**Hypothesis 12** **(H_12_).** *Millennials’ satisfaction positively influences their propensity towards recommending the fitness center.*

**Figure 1:**
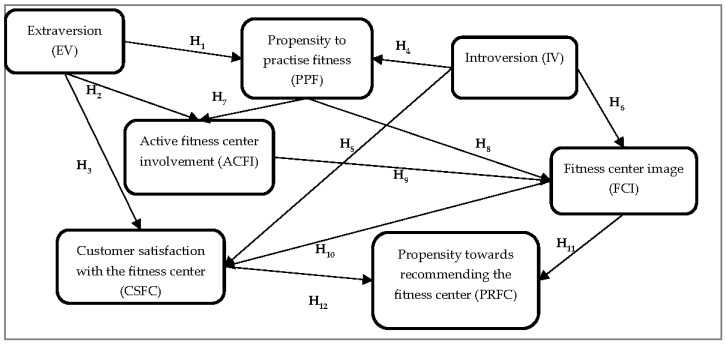
Conceptual model: The impact of extraversion and introversion on the propensity to regularly practice fitness and recommend the fitness center. Source: Own development.

Based on these arguments, the research model presented in Figure 1 was developed, containing seven constructs and twelve hypotheses. The variables proposed in the research model are deduced from the literature. Some of these have their original names: extraversion and introversion [16,17,68], the image of a fitness center [80], satisfaction [68,69,82], while others are renamed due to the need to adapt them to the items proposed in the survey questionnaire (active fitness center involvement, propensity towards recommending the fitness center). Extraversion influences customer propensity to practice fitness, active fitness center involvement, and customer satisfaction. Introversion influences consumers’ propensity to practice fitness, the fitness center image, and customer satisfaction, while the fitness center image and customer satisfaction influence the propensity towards recommending the fitness center. The propensity to practice fitness influences active center involvement and the fitness center image, while active involvement influences the fitness center image. These variables were chosen because it was considered that they have a similar relationship to those proposed by the SOR (stimulus-organism-response) model, which predicts consumer’s behavioral intentions [86]. The stimulus is the young consumer’s personality characteristics (extraversion and introversion), the organism is the consumer’s propensity to practice fitness, fitness center image, active fitness involvement, and customer satisfaction, while the response refers to the consumer’s propensity towards further recommending the fitness center to peers.

## 3. Research Methodology

### 3.1. Research Design and Sampling

To investigate Millennials’ inclination towards recommending their preferred fitness center depending on their psychological condition (introversion versus extraversion), leading to their propensity to practice fitness, their involvement with the fitness center, and their satisfaction with the fitness center, an empirical investigation employing face-to-face questionnaires was undertaken (see Figure 1). The research was conducted in 2019, with the respondents being individuals who had trained at least once during the last six months, including those who trained regularly at a fitness club. Quota sampling based on age and gender, according to the latest issue of the *Romanian Statistical Annuary*, was applied [87]. The final sample comprised only Millennials, born between 1980 and 1995 [11,12].

Out of over 3000 respondents, 2138 Millennials were considered, depending on their birth year, of whom 1064 were men (49.8%) and 1074 were women (50.2%). Most of the Millennials had been in higher education (671 men and 749 women), earned between the minimum and average wage (862 Millennials), and trained once a week (1780 individuals, 889 men and 891 women)—see Table 1. Average training lasted between 46 and 60 min (1093 people).

### 3.2. The Research Instrument

The constructs from Figure 1 (extraversion, introversion, propensity to practice fitness, etc.) were operationalized according to the international literature (see Table 2). The respondents had to assess their agreement or disagreement on a standard 5-point Likert scale towards the statements presented in Table 2. The questionnaire was initially designed in Romanian and was also implemented in Romania so that the respondents had no problems with understanding of the key concepts. The analysis procedure followed the steps recommended by the literature [88] regarding structural equation modeling in SmartPLS.

The constructs contained in the conceptual model (see Figure 1) were reflected and verified through the validity analyses and the reliability analysis (Table 2). In this regard, the collected data had to meet minimum thresholds. Table 2 shows that the item loadings exceeded the recommended threshold of 0.70, the items having convergent validity and practically measuring exactly the phenomenon under investigation [88]. Construct reliability was tested by Cronbach’s α analysis, with the values exceeding the requirement threshold of 0.7 [89]. The average variance extracted exceeded the required threshold of 0.5, highlighting the correctness of the analyzed model and the construct convergent validity. The composite reliability (CR) exceeded the threshold of 0.7, which indicates the construct’s reliability [88].

### 3.3. Analysis Procedure

Further analysis of the constructs was also performed to test the discriminant validity of the constructs. In this regard, the Fornell–Larcker and heterotrait-monotrait procedures were applied (Table 3). In the case of the Fornell–Larcker test, the diagonal values must be higher than the values below the diagonal; they represent the analysis for which the square root of the average variance was used. In the case of the HTMT test, the values must be less than 0.9, which indicates that all the concepts considered are not similar [89].

Item collinearity testing was carried out by determining the variance inflation factors for all items, with the values being below the threshold of 3.3 [89]. The highest value was 2.587 < 3.3 (PPF6 item) for the dataset, indicating there was no multi-collinearity. Next, a bootstrap procedure was applied to test the hypotheses and the relationships between the latent variables. Twelve hypotheses were accepted with a significant, positive relationship based on t-statistics.

**Figure 2:**
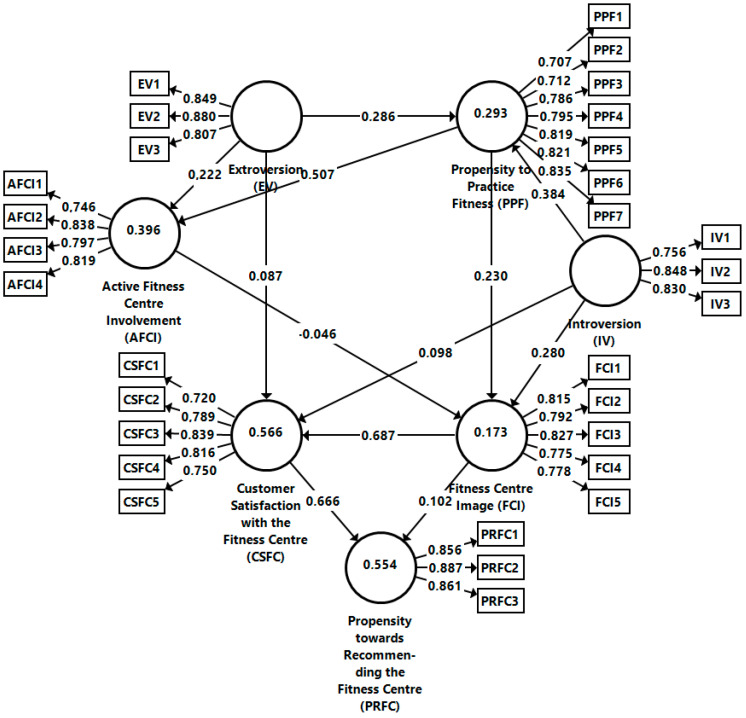
Structural model. Source: Own development in SmartPLS based on the collected data.

The dependency relations between the constructs presented in Figure 1 were investigated by structural equation modeling (SEM) using the SmartPLS software version 3.3.9 [90]. Analysis of the collinearity between the constructs highlighted the absence of this problem, with the greatest VIF value of the inner model being 2.284 (IV → FCI), significantly below the threshold of 3.3. The goodness of fit of the saturated model was also acceptable. The square root mean residual (SRMR) had a value of SRMR = 0.053 < 0.08, which met the threshold. Furthermore, extraversion, introversion, and fitness center image explained 56.6% of the variance of customer satisfaction with the fitness center (R^2^ = 0.566), while fitness center image and customer satisfaction with the fitness center explained 55.4% of the variance in propensity towards recommending the fitness center (R^2^ = 0.554), indicating the strong predictive power of the structural model (see Figure 2).

## 4. Findings

Table 4 displays the standardized regression coefficients along with their significance indicators when testing the model from Figure 1 with the bootstrap procedure. H_1_ implied that extraversion exerts a positive influence on Millennials’ propensity to practice fitness. The results (β = 0.286; T-value = 15.569; *p* < 0.001) confirmed an intense and strong positive relationship, agreeing with previous research [50], with extraversion being found to be the most important personality trait, having a major influence on individuals’ optimism [51], being higher in team sports than in individual sports [54]. Therefore, H_1_ is accepted.

H_2_ assumed that extraversion has a positive influence on active fitness center involvement. The results (β = 0.222; T-value = 12.677; *p* < 0.001) confirm the strong positive influence. Such a relationship between the abovementioned two constructs is missing in the literature and is first validated in this research. However, [19] highlighted a strong correlation between extraversion and fitness practice, while [55] demonstrated a relationship between extraversion and leisure motivation. As a result, H_2_ can be accepted. H_3_ proposed that extraversion generates Millennials’ satisfaction with the fitness center. The results (β = 0.087; T-value = 5.896; *p* < 0.001) indicate a low intensity but strong positive relation. Previous research did not demonstrate any correlation between individuals’ satisfaction and extraversion, but only between extraversion and life satisfaction [57,58], customer well-being [56,60] and life-quality [59]. Extraverts tend to be more satisfied than introverts, associated with a positive impact on customer satisfaction with the fitness center [61]. Therefore, H_3_ is partially accepted.

H_4_ implied that introversion favorably influences Millennials’ propensity to practice fitness. The results (β = 0.384; T-value = 18.956; *p* < 0.001) show an intense and strong positive relationship, which also represents a novel finding, as [63] pointed out that introverts need to live in a quiet environment. Therefore, H_4_ is accepted. H_5_ investigated the influence of introversion on Millennials’ satisfaction with the fitness center. The results (β = 0.098; T-value = 6.048; *p* < 0.001) indicate a very weak intensity but strong positive influence, not reported in the literature. Therefore, H_5_ is only partially accepted. H_6_ considered the influence of introversion on the fitness center image. The results (β = 0.280; T-value = 12.417; *p* < 0.001) indicate that the relation is of medium intensity but with a strong positive correlation, not reported in the literature. Therefore, H_6_ is accepted.

H_7_ proposed that Millennials’ propensity to practice fitness has a positive influence on their active fitness center involvement. The results (β = 0.507; T-value = 34.828; *p* < 0.001) suggest a very high influence or very strong positive significance. Past research did not validate any correlation between the propensity to practice fitness exercises and the individual’s intention to obtain information relating to their preferred sport, even though physical activity positively influences a person’s inner equilibrium [65,72]. The intensity of practicing fitness exercises when a sports club offers incentives or discounts [73] and customers’ overall satisfaction increases when they have the opportunity to obtain information by using fitness center apps [75]. So H_7_ can be accepted.

H_8_ proposed that Millennials’ propensity to practice fitness positively influences the fitness center’s image. The results (β = 0.230; T-value = 9.619; *p* < 0.001) indicate that the relationship is of medium intensity but strongly positive. This tendency is not reported in the literature, where only a correlation between the need to exercise and the negative body image of (especially) women was validated [77]. Therefore, H_8_ is to be accepted. H_9_ proposed that there would be an influence of Millennials’ active fitness center involvement on the fitness center’s image. In this case, the results (β = −0.046; T-value = 2.065; *p* < 0.05) indicate a very weak intensity and negative influence; therefore, H_9_ is partially accepted. Millennials’ active fitness center involvement decreases the perceived image of the fitness center. A correlation between the customer’s propensity to practice fitness, active fitness center involvement, and the perceived fitness center image has not been reported in the literature. H_10_ proposed that the fitness center image positively influences customer satisfaction with the fitness center. The results (β = 0.687; T-value = 45.715; *p* < 0.001) indicate a very intense and strong positive effect, in accordance with the literature [81,82]. So, the tenth hypothesis is confirmed.

H_11_ proposed the influence of fitness center image on the propensity towards recommending the fitness center. The results (β = 0.102; T-value = 4.645; *p* < 0.001) indicate a relationship that is weak in intensity, but strongly positive. Similar findings regarding customers’ future intentions toward sports activities were reported by [80]. Furthermore, commitment to a sport’s brand positively influences its recommendation to other potential customers [84]. Therefore, H_11_ is accepted. The last hypothesis concerned the influence of customer satisfaction on the fitness center and on the propensity towards recommending the fitness center. The results (β = 0.666; T-value = 33.500; *p* < 0.001) indicate a high intensity and strong positive relation, similar to previous research [83,85]. Therefore, H_12_ is to be accepted.

## 5. Discussion

### 5.1. Theoretical Implications

A fitness center is visited by both extraverts and introverts, regardless of the generation to which they belong. Considering Millennials’ specific features that distinguish them from other generational cohorts (altruistic, optimistic, protective, and consumeristic orientation), the factors linking extraversion and introversion to customer satisfaction, which ultimately generate the intention to recommend the preferred fitness center to peers, were analyzed in the present research. From a theoretical perspective, the paper has a multidisciplinary character, its theme extending to fields such as sport, marketing, psychology, health, and sociology. The research adds value to generational theory (also referred to as the sociology of generations), extending the understanding of Millennials’ desires and involvement in sports activities and fitness practices. The novelty of the paper consists in assessing how Millennials’ fitness center involvement depends on their personality type, as well as their perceived image of the fitness center and their involvement in regular training. Extraverted or introverted Millennials’ impact on the present and future effectiveness of a fitness center is important in keeping the fitness business sustainable.

Another novelty is that the research defines for the first time in the specialized literature the terms active fitness center involvement, fitness center image, and positive behavioral intention in the context of a fitness center. Active fitness involvement influences the image of the fitness center, which impacts customer satisfaction and might also influence the propensity to recommend the fitness services to other potential customers. Some of the formulated hypotheses are slightly modified in the literature (H_1_, H_3_, H_7_, H_10_, H_11_, H_12_), while the others are novel, being defined and validated for the first time in our paper (H_2_, H_4_, H_5_, H_6_, H_8_, H_9_). The proposed constructs are inspired by the literature, having the original name (extraversion, introversion, fitness center image, satisfaction with a fitness center), or a slightly modified one adapted to the proposed items (active fitness center involvement, propensity towards recommending the fitness center). The variables were chosen based on the stimulus–organism–response (SOR) model from environmental psychology, with extraversion and introversion being the stimuli, the consumer’s propensity to practice fitness, fitness center image, active fitness involvement, and customer’s satisfaction being the organism, and the consumers’ propensity towards further recommending the fitness center to peers being the response. All the proposed hypotheses were confirmed.

The research revalidated the presumption that extraversion positively influences customers’ propensity to practice fitness exercises [50,51], but validated for the first time a relationship between extraversion and active fitness involvement. The presumption that extraversion influences customers’ satisfaction is only partially accepted, agreeing with [61] in showing that extraverts are more satisfied than introverts. This observation is similar to the results suggested in the psychological model [53] in accordance with which extraversion increases an individual’s well-being, while introversion decreases it. Although this research has as its starting point psychological theories, such as those of Jung and Myers–Briggs, as well as generational theory from sociology, the surprising fact is that their observations generate new interpretations when they are applied, for example, in the field of sports in an emerging market, as in this case. These differences are noticeable primarily in the behavior of introverts when it comes to their preferred fitness center. The research demonstrated that being a Romanian introvert favorably influences a customers’ propensity to practice fitness, which is contrary to the suggestion in [63], arguing the opposite: introverted people like to live in a quiet environment. Furthermore, introversion positively influences the fitness center image; the relationship is validated for the first time in this research. It was also found that there is a connection between introversion and customer satisfaction, a relationship validated for the first time in this work. The results of the survey show that Millennials tend to train regularly at a fitness center. This behavior leads us to infer that Romanian Millennials, regardless of whether they are introverted or not, like to exercise regularly and join a fitness center. We consider that these interesting results represent a contribution to the marketing literature.

The propensity to practice fitness positively influences active fitness involvement [73] and the fitness center image. The proposed relationship between Romanian Millennials’ active involvement and the fitness center image is partially accepted. The influence of fitness center image on peer recommendation is revalidated [78,82]. Finally, there is a link between Millennials’ satisfaction and their propensity to recommend a fitness center to peers [83,85].

Extraversion positively influences Millennials’ active fitness center involvement, the propensity to practice fitness, and satisfaction. Introversion influences the fitness center image, the propensity to practice fitness, and customer satisfaction [91]. There is a negative relationship between Millennials’ active fitness center involvement and the perceived fitness center image [92], while the propensity to practice fitness positively influences Millennials’ active fitness center involvement and the fitness center image. The fitness center image influences Millennials’ satisfaction [93] and customers’ propensity to recommend a fitness center, and finally, Millennials’ satisfaction [94] influences their propensity to recommend the fitness center to other individuals [95].

The present research shows that extraverts are more interested in gathering new information regarding their favorite sport and feel satisfied when they are regularly practicing fitness, as was also stated previously [5]. But introversion also favorably influences Romanian Millennials’ satisfaction with the services offered by a fitness center (hypothesis H_4_ was validated for the first time in the present research). Surprisingly, the survey shows that, despite introverts closed psychological profile, Romanian introvert Millennials still have a high inclination to practice fitness. At the same time, a positive image of the fitness center stimulates the satisfaction of extraverts and introverts, consistent with previous findings [80,82] that indicate that this variable further influences the consumer’s intention to practice fitness [50] and to recommend the preferred fitness center to peers [83].

### 5.2. Managerial Implications

The management of a fitness center must take into consideration that in the last few years the extension of the fitness industry has attracted the interest of new investors, while young generations, such as Millennials, interested in a healthy lifestyle, are consuming sports differently from their parents. Because of market competition, fitness centers are working hard to attract new clients. To have success in the modern market and to satisfy the client’s expectations in the best way, fitness centers must adapt to a variety of human personalities. In this context, from a managerial perspective, the research highlights to fitness center managers the relevance of customers’ personality types (extraverts and introverts) and their propensity to practice fitness, also showing the impact of sports exercise in generating customer satisfaction and on customers’ inclination towards recommending their preferred fitness club to peers. Managers who are truly interested in accommodating both types of personalities should consider offering a wide range of options regarding workout activities.

From the point of view of the personality profile of the Millennials and from that of the marketing strategies adopted by a fitness center, and due to the need and desire of Generation Y to receive supplementary information related to their favorite fitness center, the management of these businesses must implement strategies to improve customer relations and use modern means to promote the offered services (incentives, mobile apps, etc.). Extraverted and introverted Millennials will be able to achieve a higher level of satisfaction if the manager of the center improves the price and promotion strategies, thus strengthening the client–employee relationship, and, in turn, the client–company relationship, which will ultimately lead to customer loyalty. In this context, as the results of this research show, and considering that Millennials are conscious about their health and fitness practices, a fitness center should offer financial facilities to clients to keep present customers, further attract new customers, and influence whether they will further recommend the center to peers.

As modern consumers have less free time and tend to adopt a predominantly sedentary lifestyle, fitness centers should offer quality service packages that suit all preferences, regardless of individual personality types. Fitness centers must make consistent efforts to attract new customers, as their future success depends not only on how satisfied their customers are but also on their predisposition to further recommend fitness services to peers. As a practical implication, it is recommended for the fitness center management to make efforts to create a suitable ambience where both introverts and extraverts can be comfortable. Since the social nature of Romanian extravert Millennials seems to be dynamic, although introverts are enthusiastic, introverts must feel more secure while practicing sports, which is why a better communication strategy that creates a closer relationship between the customers (extraverts and introverts) and the employees and clients is needed. To achieve this goal, the organization can arrange birthday celebrations for employees, where customers can also be invited. Additionally, reward actions can be implemented for loyal customers, along with bonuses for clients who recommend the center and attract new clients. Furthermore, group training sessions can be conducted to facilitate the integration of introverted clients.

Furthermore, considering the unprecedented development of AI, fitness club managers need to adapt their businesses to keep up with the pace of digital technology. To differentiate the services offered to extraverted and introverted clients and to better adapt to each personality type, it is recommended that managers provide virtual assistants (e.g., chatbots), improving in that way the quality of the services offered to the clients. Managers should prioritize instructors’ competence, maintain well-equipped and varied fitness facilities, ensure cleanliness, and align service value with customer expectations to enhance satisfaction. Notably, attributes tied to instructors’ performance, fitness equipment, overall cleanliness, and the value of service emerge as focal points [96]. Interpersonal interactions, including courtesy and prompt service, significantly shape customer perceptions [97]. Enhancing service quality, especially through employee commitment and individualized treatment, is crucial for customer satisfaction and long-term relationships, increasing the likelihood of membership renewal [98].

Finally, it should be mentioned that an efficient way to promote the fitness center is to improve its future image. With increased customer recognition of the offered services, the image determines the attitude and future behavior of Millennials in recommending their favorite fitness center to others.

### 5.3. Limitations and Future Research Perspectives

This analysis has a complex character and encompasses several fields (sport, marketing, psychology, health, and sociology), which is why it is multidisciplinary. As an extensive analysis of all these fields was not possible, the research has certain limits.

The limitations of our study include the fact that the analysis focused only on Generation Y, and not on other generations, like Baby Boomers, Xers, or Zers. The research refers only to practicing sports in fitness centers, which is why it would not be possible to extrapolate the findings to other sports fields. Therefore, future research could focus on the behavior and perceptions of different customer generations when training in fitness centers (or practicing any kind of sport) and their propensity towards recommending the fitness center (the sports service) on social media. It would also be interesting to highlight the impact of socio-economic events on the propensity to practice fitness or to investigate the impact of word-of-mouth on individuals’ training habits. The quota sampling method that we employed to gather data for this investigation is another important limitation of our investigation. There is always the chance of sampling bias because survey randomization is not taken into consideration when using this non-probability sampling approach, which depends on the non-random selection of a predefined number or proportion of respondents. Therefore, future studies could use probability sampling techniques, such as stratified sampling, to investigate these relationships.

Another limitation concerns the fact that although in Figure 1 there are lots of mediations, these relations have not been analyzed. The mediation effects are also equally important, but as the proposed model contains quite a large number of mediation effects, only the direct effects have been considered. It was of the utmost importance to highlight the influence of introversion and extraversion on the image of the fitness center and the propensity of Millennials to recommend their preferred fitness center. Future studies should, of course, also address relevant mediation effects, such as those between the fitness center image and fitness center satisfaction and their impact recommendations for a preferred fitness center.

## 6. Conclusions

This research aimed to analyze the impact of Millennials’ personalities (extraverts and introverts) on their decision when, or if, to recommend their preferred fitness center in an emerging country. Both personality features influence not only customers’ propensity to practice fitness exercises but also their satisfaction with a fitness club.

Extraversion and introversion influence Millennials’ satisfaction with the services a fitness center offers. Apart from the potential differences between extraverted and introverted Millennials, the research revealed that the propensity to practice fitness depends on each individual, whether training alone or in teams. In this respect, fitness centers could tailor their offers to Millennials’ expectations.

## Figures and Tables

**Table 1:** The socio-demographic characteristics of the considered sample.

		Male		Female		Total	
		%	n	%	n	%	n
Studies	High-school studies	324	15.2	273	12.8	597	27.9
	Post-secondary/professional	69	3.2	52	2.4	121	5.7
	Higher	671	31.4	749	35.0	1420	66.4
	Total	1064	49.8	1074	50.2	2138	100.0
Income	Below the minimum wage	58	3.8	88	5.8	146	9.6
	Between minimum and average wage	419	27.6	443	29.2	862	56.7
	Double the average salary	233	15.3	209	13.8	442	29.1
	Four average salaries and above	53	3.5	16	1.1	69	4.6
	Total	763	50.2	756	49.8	1519	100.0
Workouts	One workout per week	889	41.6	891	41.7	1780	83.3
	Two training sessions per week	39	1.8	68	3.2	107	5.0
	Three training sessions per week	77	3.6	67	3.1	144	6.7
	Daily workouts	59	2.8	48	2.2	107	5.0
	Total	1064	49.8	1074	50.2	2138	100.0
Training time	up to 30 min	60	2.8	45	2.1	105	4.9
	31–45 min	59	2.8	72	3.4	131	6.1
	46–60 min	466	21.8	627	29.3	1093	51.1
	61–90 min	307	14.4	197	9.2	504	23.6
	91–120 min	147	6.9	116	5.4	263	12.3
	up to 120 min	25	1.2	17	0.8	42	2.0
	Total	1064	49.8	1074	50.2	2138	100.0

Source: own development based on the collected data.

**Table 2:** Constructs and Items.

Construct	Item	Description	Loading	α/CR/AVE
Extraversion (EV) adapted after [20,54,58,59].	EV1	Training at the gym is a good opportunity to meet friends/acquaintances.	0.849	0.801/0.883/0.716
	EV2	I prefer training at the gym because I can socialize with other athletes.	0.880	
	EV3	I prefer training at the gym because I like to train with others.	0.807	
Introversion (IV) adapted after [63].	IV1	Working out at the gym makes me feel strong.	0.756	0.742/0.853/0.660
	IV2	Training at the gym gives me strength.	0.848	
	IV3	I feel good when I work out in a gym.	0.830	
Propensity to Practice Fitness (PPF) adapted after [73].	PPF1	Fitness is a fun activity for me.	0.707	0.894/0.917/0.614
	PPF2	I enjoy discussing fitness with my friends and family.	0.712	
	PPF3	I am an avid fitness fan.	0.786	
	PPF4	Of all the sports I practice, I prefer fitness.	0.795	
	PPF5	Fitness is an important sport for me.	0.819	
	PPF6	Fitness is a sport that interests me.	0.821	
	PPF7	Fitness is a sport that fully satisfies me.	0.835	
Active fitness center involvement (AFCI) adapted after [75,76].	AFCI1	I consider myself a true professional when I think about training in the gym.	0.746	0.813/0.877/0.641
	AFCI2	I enjoy reading articles on types of fitness training.	0.838	
	AFCI3	I watch TV shows that show how to train.	0.797	
	AFCI4	I regularly read fitness magazines.	0.819	
Fitness Center Image (FCI) adapted after [78,80].	FCI1	The package I benefit from is exactly as I expected.	0.815	0.857/0.897/0.636
	FCI2	The package I benefit from is provided as promised (in advertising, etc.).	0.792	
	FCI3	My expectations regarding training at my favorite gym were met.	0.827	
	FCI4	I generally trust my favorite gym.	0.775	
	FCI5	I am satisfied with the quality/price ratio of the services received at the fitness club.	0.778	
Customer Satisfaction with the Fitness Center (CSFC) adapted after [81,82].	CSFC1	I am satisfied with the training so far.	0.720	0.842/0.888/0.615
	CSFC2	My decision to choose this fitness center was a wise one.	0.789	
	CSFC3	I enjoy working out in this gym.	0.839	
	CSFC4	I do not regret training in this gym.	0.816	
	CSFC5	I will tell my friends and family how satisfied I am with this gym.	0.750	
Propensity towards recommending the fitness center (PRFC) adapted after [80,83,84].	PRFC1	I will recommend the trainers of the fitness club to my friends/acquaintances.	0.856	0.837/0.902/0.754
	PRFC2	In the future, I will also recommend training at my gym to others.	0.887	
	PRFC3	I can positively present my favorite gym to my relatives or friends.	0.861	

Note: Factor loading > 0.6; Cronbach’s Alpha/α > 0.7; Average variance extracted (AVE) > 0.5; Composite reliability (CR) > 0.7 [88].

**Table 3:** Discriminant validity analyses.

Construct	AFCI	CSFC	EV	FCI	IV	PPF	PRFC
Fornell–Larcker Criterion							
AFCI	0.801						
CSFC	0.225	0.784					
EV	0.424	0.238	0.846				
FCI	0.180	0.739	0.179	0.798			
IV	0.318	0.380	0.294	0.373	0.812		
PPF	0.596	0.362	0.399	0.333	0.468	0.784	
PRFC	0.232	0.741	0.239	0.594	0.306	0.292	0.868
Heterotrait-Monotrait Criterion							
AFCI							
CSFC	0.268						
EV	0.525	0.290					
FCI	0.211	0.868	0.217				
IV	0.410	0.477	0.385	0.461			
PPF	0.687	0.417	0.467	0.381	0.573		
PRFC	0.279	0.877	0.292	0.698	0.387	0.336	

Note: AFCI: Active fitness center involvement; CSFC: Customer satisfaction with the fitness center; EV: Extraversion; FCI: Fitness center image; IV: Introversion; PPF: Propensity to practice fitness; PRFC: Propensity towards recommending the fitness center. Source: Own development based on the collected data.

**Table 4:** The path coefficients of the structural equation model.

Paths	Path Coefficients	Standard Deviation	T-Value	*p*-Value	Hypotheses
EV → PPF	0.286	0.018	15.569	0.000 ***	H_1_-Accepted
EV → AFCI	0.222	0.018	12.677	0.000 ***	H_2_-Accepted
EV → CSFC	0.087	0.015	5.896	0.000 ***	H_3_-Accepted
IV → PPF	0.384	0.020	18.956	0.000 ***	H_4_-Accepted
IV → CSFC	0.098	0.016	6.048	0.000 ***	H_5_-Accepted
IV → FCI	0.280	0.023	12.417	0.000 ***	H_6_-Accepted
PPF → AFCI	0.507	0.015	34.828	0.000 ***	H_7_-Accepted
PPF → FCI	0.230	0.024	9.619	0.000 ***	H_8_-Accepted
AFCI → FCI	−0.046	0.022	2.065	0.039 *	H_9_-Accepted
FCI → CSFC	0.687	0.015	45.715	0.000 ***	H_10_-Accepted
FCI → PRFC	0.102	0.022	4.645	0.000 ***	H_11_-Accepted
CSFC → PRFC	0.666	0.020	33.500	0.000 ***	H_12_-Accepted

Note: * *p* < 0.05; *** *p* < 0.001; AFCI: Active fitness center involvement; CSFC: Customer satisfaction with the fitness center; EV: Extraversion; FCI: Fitness center image; IV: Introversion; PPF: Propensity to practice fitness; PRFC: Propensity towards recommending the fitness center. Source: Own development based on the collected data.

## Data Availability

Data will be made available on request from the corresponding author.
